# Antibiotic Use and Stewardship Indicators in the First- and Second-Level Hospitals in Zambia: Findings and Implications for the Future

**DOI:** 10.3390/antibiotics11111626

**Published:** 2022-11-15

**Authors:** Aubrey C. Kalungia, Moses Mukosha, Chiluba Mwila, David Banda, Matthews Mwale, Solomon Kagulura, Olanyika O. Ogunleye, Johanna C. Meyer, Brian Godman

**Affiliations:** 1 Department of Pharmacy, University of Zambia, Lusaka P.O. Box 50110, Zambia; 2 Department of Nursing, Chreso University, Lusaka P.O. Box 37178, Zambia; 3 Department of Clinical Care & Diagnostic Services, Ministry of Health, Lusaka P.O Box 30205, Zambia; 4 The World Bank, Zambia Country Office, Lusaka P.O Box 35410, Zambia; 5 Department of Pharmacology, Therapeutics and Toxicology, Lagos State University College of Medicine, Lagos 100271, Nigeria; 6 Department of Medicine, Lagos State University Teaching Hospital, Lagos 100271, Nigeria; 7 Department of Public Health Pharmacy and Management, School of Pharmacy, Sefako Makgatho Health Sciences University, Pretoria 0204, South Africa; 8 Centre of Medical and Bio-Allied Health Sciences Research, Ajman University, Ajman 346, United Arab Emirates; 9 Department of Pharmacoepidemiology, Strathclyde Institute of Pharmacy and Biomedical Sciences, University of Strathclyde, Glasgow G4 0RE, UK

**Keywords:** antibiotic use, antimicrobial stewardship programmes, AWaRe classification, guidelines, point prevalence survey, quality indicators, Zambia

## Abstract

**Introduction:** There are increasing concerns with growing rates of antimicrobial resistance (AMR) across Africa, including in Zambia, enhanced by inappropriate utilization of antibiotics across the sectors. There is a need in hospitals to document current prescribing patterns via point prevalence surveys (PPS) alongside recognized indicators to improve future use. The findings can subsequently be used to develop and instigate appropriate antimicrobial stewardship programs (ASPs) to improve the quality of future antimicrobial prescribing across Zambia. This includes encouraging the prescribing of ‘Access’ over ‘Watch’ and ‘Reserve’ antibiotics where pertinent. **Methods:** A PPS was undertaken using the WHO methodology among 10 first- and second-level public hospitals across the 10 provinces of Zambia. A sampling process was used to select the hospitals. **Results:** The prevalence of antibiotic use among the in-patients was 307/520 (59.0%), with a high rate of empiric prescribing of ceftriaxone at 36.1% of all antibiotics prescribed (193/534). The reason for antibiotic use was recorded in only 15.7% of occasions and directed treatment prescribed in only 3.0% of occasions. Compliance with the national standard treatment guidelines (STGs) was also low at only 27.0% of occasions. **Conclusion:** High empiric prescribing, limited documentation of the rationale behind antibiotic prescribing, high use of ‘Watch’ antibiotics, and limited compliance to STGs among surveyed hospitals requires the urgent instigation of ASPs across Zambia to improve future prescribing.

## 1. Introduction

Antimicrobial resistance (AMR) is increasingly seen globally, with an estimated 4.95 million deaths associated with bacterial AMR in 2019, which includes 1.27 million deaths directly attributable to AMR [1,2]. The greatest burden of AMR is currently seen in Sub-Saharan Africa, with deaths from AMR globally potentially reaching more than 10 million annually by 2050 if not addressed [1]. There are also considerable costs associated with AMR, potentially up to 3.8% of annual global gross domestic product (GDP) unless addressed [3,4,5].

The concerns with growing morbidity, mortality, and costs associated with AMR have resulted in an appreciable number of global, regional, and national initiatives. These include the development of the Global Action Plan to reduce AMR by the World Health Organization (WHO) as well as activities among the Organisation for Economic Co-operation and Development (OECD) Health Policy Group, the Interagency Coordination Group on Antimicrobial Resistance (ICGAR) group, and the World Bank [6,7,8,9,10]. The WHO Global Action Plan to reduce AMR resulted in the development of National Action Plans (NAPs) across continents including those from across Africa [11]. Zambia is no exception launching its NAP in 2017, which involved all key stakeholder groups [11,12]. This includes a multisectoral national Antimicrobial Resistance Coordinating Committee (AMRCC) on AMR. Ongoing activities across Zambia also include documenting current antimicrobial use in hospitals, the prescribing of antibiotics in ambulatory care, the procurement of antibiotics without a prescription, and extensive veterinary use, including among poultry farmers, given the potential for AMR with their overuse [13,14,15,16]. There have also been concerns with the management of patients with community-acquired pneumonia in hospitals in Zambia with high mortality rates, enhanced by high co-morbidity rates of the human immunodeficiency virus (HIV) and tuberculosis (TB) among patients [17].

Within hospitals, point prevalence surveys (PPS) are a well-established methodology to measure current antibiotic utilization patterns in order to develop targets for quality improvement and antimicrobial stewardship programs (ASPs) [13,18,19,20]. Potential targets for ASPs include measures to increase culture and sensitivity testing, reduce extended antibiotic prophylaxis to prevent surgical site infections (SSIs), encourage de-escalation to oral antibiotics, and enhance adherence to current guidelines [18,21,22]. Initially, there were concerns regarding the ability of low- and middle-income countries to undertake ASPs due to resource issues in both personnel and available funds [23]. In addition, concerns with knowledge regarding ASPs among key hospital personnel [24,25]. However, this is changing with ASPs increasingly being undertaken among African countries to good effect, including reducing extended prophylaxis to prevent SSIs [26,27,28,29,30,31,32,33]. More recently, there have been moves to assess current antimicrobial prescribing based on the WHO AWaRE categorization as targets for quality improvement programmes to reduce the unnecessary prescribing of ‘Watch’ and ‘Reserve’ antibiotics and their associated resistance potential [21,34,35,36]. This initiative is likely to stay given concerns with rising AMR rates across countries and continents including Sub-Saharan Africa and the need to focus the minds of all key stakeholder groups on critical areas of prescribing [1,34,35,36,37,38].

We are aware that a few PPS studies have taken place in some tertiary level hospitals in Zambia, demonstrating concerns with antimicrobial prescribing patterns [13,14]. In the existing literature, there do not appear to be any PPS studies that have been undertaken among lower-level hospitals in Zambia, including district and provincial hospitals where patients can be referred from health centers and clinics, to identify potential targets for ASPs to improve future prescribing. Consequently, we wanted to address this, especially given the identified concerns with antimicrobial prescribing among tertiary hospitals in Zambia [13,14]. In addition, build on recent activities to enhance ASPs and reduce AMR within hospitals in Zambia as part of the ongoing NAP [11]. This was the aim of this study.

## 2. Results

### 2.1. Frequency and Demographics of Patients Prescribed Antibiotics

Out of 520 patients surveyed among the 10 public hospitals across the 10 provinces of Zambia during November 2021, a total of 307 in-patients were treated with a total number of 534 antibiotics. This gives a 59.0% pooled prevalence of antibiotic use, ranging from 30.0% to 79.4% of the 520 in-patients surveyed across the 10 participating hospitals. Amongst the 307 patients who were prescribed antibiotics, an average number of 1.7 (SD: 0.75) antibiotics was prescribed per patient.

Table 1 documents the demographics of the 307 patients who were prescribed antibiotics across the surveyed hospitals, including the ward specialty where the antibiotics were prescribed, their ages, gender, extent of catheterization, and extent of other infectious diseases, including HIV, malaria, and TB.

Among the 520 patients surveyed, in terms of the types of wards where antibiotics were most prescribed, pediatric as compared to adult wards had a higher rate, with the Neonatal Intensive Care Unit (NICU) having the highest use at 20 patients amounting to 83.3% of those in the NICU at the time of the survey. The lowest pooled use was in the mixed pediatric wards at 47.7% (overall 27 patients among the 10 hospitals). This compares with rates of 49.7% to 52.5% among patients in the adult wards (overall 189 patients). For 17 patients who were prescribed antibiotics, their ward location was not recorded.

### 2.2. Nature of Antibiotics Prescribed including Infections

Table 2 provides details of the commonly diagnosed infections that were treated using antibiotics among the 10 surveyed hospitals. The most common infections were obstetrics and gynecological infections (12.4%), followed by pneumonia (11.7%). Ceftriaxone—a ‘Watch’ antibiotic—was the most frequently prescribed antibiotic first line across the diagnosed infections, accounting for 193 of the 534 antibiotics (36.1%) among the 307 patients prescribed antibiotics across the surveyed hospitals. This was followed by cefotaxime—also a ‘Watch’ antibiotic—with 70 prescriptions (13.1%). The least prescribed of the first eight commonly prescribed antibiotics was gentamicin—an ‘Access’ antibiotic—in 25 cases (4.7%). Most (76.2%) antibiotics were prescribed for intravenous use. The mean number of missed doses was 1 (SD = 1). Overall, 209 missed doses were identified for the 534 antibiotics prescribed. However, the reasons were not typically known as these were not documented in the notes (189/209, 90.4%). Where documented, the reasons included ‘delayed initiation of the prescribed medication’, ‘patient was away from the ward at administration time’, and ‘the medication was not available from the pharmacy/out of stock (6/209, 2.9%)’.

### 2.3. Commonly Prescribed Antibiotics among Surveyed Hospitals

**Figure 1:**
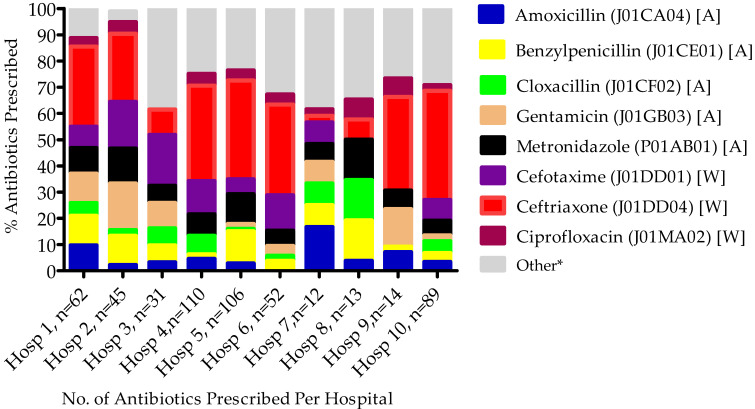
Commonly prescribed antibiotics at the hospital level. Key: * Other antibiotics prescribed include ampicillin, azithromycin, co-trimoxazole, erythromycin, meropenem, nitrofurantoin, and penicillin-V; A = Access, W = Watch. Patients could be prescribed more than one antibiotic.

Figure 1 shows the commonly prescribed antibiotics at the hospital level among the 10 participating hospitals, with variation among the hospitals depending on the nature of the public hospital and the patients’ diagnoses. No ‘Reserve’ antibiotics were prescribed.

### 2.4. Commonly Prescribed Antibiotics at the Ward Level among Surveyed Hospitals

**Figure 2:**
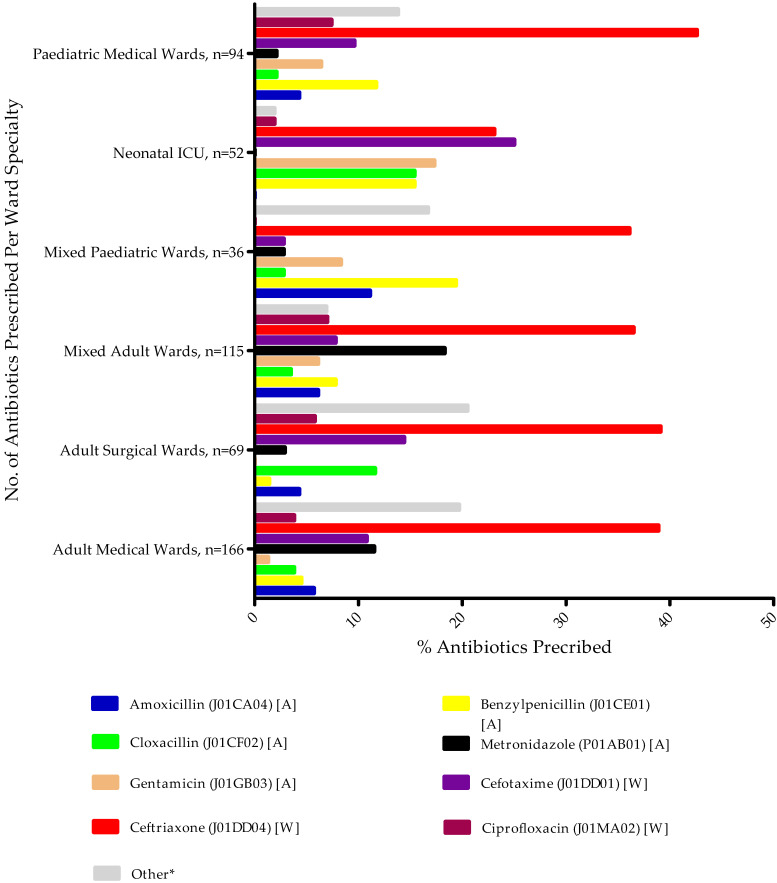
Commonly prescribed antibiotics at the ward level among participating hospitals. Key: * Other antibiotics prescribed include ampicillin, azithromycin, co-trimoxazole, erythromycin, meropenem, nitrofurantoin, and penicillin-V; [A]: Access; [W]: Watch. Patients could be prescribed more than one antibiotic.

Figure 2 shows the most commonly prescribed antibiotics at the ward level, again distributed by ATC and AWaRe classification, with no antibiotics prescribed from the ‘Reserve’ group. The most prescribed antibiotic in Adult Medical Wards was ceftriaxone, accounting for 61 (38.9%) of occasions, and the least out of the most frequently prescribed antibiotics was gentamicin (1.3%). Similarly, in the Adult Surgical Wards, Mixed Adult Wards, Mixed Pediatric Wards, Neonatal Intensive Care Unit, and Pediatric Medical Wards, respectively, the most prescribed antibiotic was ceftriaxone, accounting for 27 (39.1%), 42 (36.5%), 13 (36.1%), 12 (23.1%), and 40 (42.6%) of occasions, respectively. The least prescribed antibiotic among the top eight varied by ward. This was benzylpenicillin in the Adult Surgical Wards, cloxacillin in the Mixed Adult Wards, and a mixture in the Mixed Pediatric Wards, Neonatal Intensive Care Units, and Pediatrics Medical Wards.

### 2.5. Antibiotic Prescribing Broken down by Key Quality Indicators

Table 3 shows the quality of prescribing among the surveyed hospitals distributed by key indicators. There were concerns with the limited recording of the rationale for the antibiotics being prescribed (*n* = 534) in patients’ notes among the surveyed hospitals (15.7%), which varied from 48.9% of occasions for community-acquired infections to only 2.1% for medical prophylaxis. There was also limited use of culture and sensitivity testing (CST) to guide antibiotic choices (3%), which resulted in correspondingly high rates of empiric prescribing (97%). In addition, there were concerns with limited documentation of review and stop dates for the antibiotics prescribed. This was in addition to the limited recording of the rationale for missed doses of antibiotics in patients’ notes.

Compliance with the Zambian national standard treatment guidelines (STG) was seen in 27.0% of cases, highest among patients in the mixed wards (44.9%) but lowest in ICU wards at 1.3%, which is a concern. In addition, highest for community-acquired infections.

Encouragingly, there was a high rate (83%) of international non-proprietary name (INN–generic) prescribing (Table 3).

## 3. Discussion

We believe this is the first comprehensive PPS study undertaken in Zambia to provide baseline data regarding current antibiotic use patterns among both first- and second-level public hospitals in the 10 provinces in Zambia. The infections seen reflect patients typically treated in these hospitals versus those treated in tertiary hospitals in Zambia. In addition, provide key areas to inform future quality improvement programs and capacity development of ASPs in Zambia. The latter is important as there have been concerns in Zambia with knowledge regarding ASPs among clinicians and pharmacists even in tertiary hospitals [24]. The overall prevalence of antibiotic use was 59.0% among the 10 participating public hospitals across Zambia, with all but 2 hospitals recording a prevalence above 40% (higher than the 40% threshold recommended by the WHO) [39]. The documented prevalence rate for antibiotic use among the surveyed hospitals in Zambia is higher than seen in South Africa (37%–49.7%) [40,41], among the consolidated findings of the 303 hospitals from 53 countries taking part in the Global PPS at 34.4% [18], as well as among America, European and Oceanian hospitals in the systematic review of Saleem et al. (2020), where prevalence rates averaged 32.5% to 38.9% [20]. The prevalence seen among these hospitals in Zambia was, however, similar or lower than among the consolidated African hospitals in the systematic review of Saleem et al. (2020) at 62.7% [20] as well as among hospitals in Ghana (54.9%–82%) [19,42,43,44], Kenya (52.0%–67.7%) [45,46,47], Tanzania (62.3%) [48] alongside 17 hospitals across Ghana, Uganda, Zambia, and Tanzania [13]. The prevalence rates in Zambia were also lower than seen in Botswana (70.6%) [49], Eswatini (88.2%) [50], Nigeria (76.6% to 80.6%) [22,51,52], and Uganda (74%) [21].

Ceftriaxone, a ‘Watch’ list antibiotic, was the most prescribed antibiotic across the 10 surveyed hospitals in Zambia, accounting for 36.1% of first antibiotic prescriptions. This is similar to the systematic review of Saleem et al. (2020), where third-generation cephalosporins were among the most prescribed antibiotics [20]. The prescribing rate was also similar to or higher than seen in Eswatini (21.6%) [50], Kenya (29% to 39.7% of prescriptions in two hospitals with third-generation cephalosporins accounting for 55% of antibiotics prescribed in another) [45,46,47], Nigeria (13.7%–37%) [22,51,52], Tanzania (30.9%–49%) [48], and Uganda (37%–44%) [13,21]. There was also appreciable prescribing of ceftriaxone among hospitals in South Africa although at a lower rate (10.7% based on DDDs) [40]. Similar to D’Arcy and colleagues [13], encouragingly there was no prescribing of ‘Reserve’ antibiotics among the 10 participating hospitals, unlike other hospitals in Africa [36]. This is particularly important given that patients from first- and second-level hospitals can be transferred to tertiary hospitals for more specialized care if needed, and inappropriate prescribing of ‘Reserve’ antibiotics outside of specialist hospitals reduces their potential to tackle serious and critical infections [36,53]. Our findings are similar to the low or no prescribing of ‘Reserve’ antibiotics among other African countries in published PPS studies [21,36,42,43,46,48,49,50,51,54], which is also encouraging.

Of concern is that the high rates of ceftriaxone prescribing among the hospitals in Zambia have resulted in resistance rates as high as 90% among some hospitals in Zambia, alongside 80% resistance to ciprofloxacin and 70% resistance to gentamicin [25,55]. AMR is fueled by high rates of empiric therapy (97% in this study), which needs to be addressed going forward.

There were also concerns regarding adherence to key quality indicators among the participating hospitals in Zambia (Table 3), with compliance rates considerably lower than seen among the African countries in the Global PPS (67.9%) and across the 53 countries (77.4%) [18]. Key issues to address in future ASPs in Zambia include improved recording of the indication for which the antibiotics are being prescribed in the patients’ notes, with these details currently being recorded in only 15.7% of occasions. This is appreciably lower than seen in the study of D’Arcy et al., where the indications for antibiotic prescriptions being recorded in patients’ notes ranged from 66% to 100% of all prescriptions in Ghana and up to 97% in Uganda [13,42], as well as among the African countries participating in the Global PPS study (70.4%), overall (76.9%) [18], and appreciably lower than seen in a recent study in South Africa where less than 6% of in-patient antibiotic prescriptions had no indication recorded [54].

There is also a need to increase CST and antibiogram data use to address concerns with current extensive empiric prescribing. The low rate of CST requests seen among the participating hospitals (3% of occasions) may be due to current limited capacity among the first- and second-level public hospitals to undertake any CST analyses, with CST requests potentially sent to higher hospitals or private laboratories for analysis. This is similar to the situation among a range of hospitals in South Africa, which results in delays in reporting the results impacting on requests in practice [56,57]. The current rate of 3% was appreciably lower though than seen in some other African countries [47,58], as well as among participating hospitals in the Global PPS study (targeted treatment in 19.8% of occasions) [18]. However, Kiggundu et al., in their study in Uganda, reported that no patients were treated based on CST results [21], similar to Eswatini [50], with very low rates for CST requests also seen in Tanzania and Ghana [43,48]. To improve CST requests will require investment in laboratory capacity, materials, and relevant skilled staff in Zambia, building on commitments made in the Zambian NAP [11]. We will be following this up as Zambia continues to build ASP capabilities to improve future antimicrobial prescribing in its hospitals.

There is also concern that compliance with the Zambia NSTGs was low at only 27.0% of occasions, which, as mentioned, was appreciably lower than seen in the consolidated findings in the Global PPS study (77.4%) as well as among participating African countries (67.9%) [18]. In addition, it was lower than that seen among a number of other African countries, including Ghana (50.0%–66.7%) [42], Kenya (45.8%) [46], South Africa (90.2%) [54], Tanzania (50%–63%) [25,59], Uganda (29.7%–30.9%) [21], and Zimbabwe among children (57.7%) [60]. This low level of compliance to guidelines in Zambia may be due to concerns about irregular updating of guidelines and if these are being adapted from high-income countries without local knowledge and input [14]. Alongside this are issues around implementation and availability of STGs in the local hospitals, with similar concerns highlighted in Botswana [49]. Greater adherence to guidelines can be achieved by being up to date and easy to use [61,62,63]. Future Zambian NSTGs must also reflect current recommendations in the WHO AWaRe list and be informed by local sensitivity data. We will also be following this up in the future. Where treatment guidelines are implemented and routinely monitored, compliance levels are typically higher. For instance, in South Africa, Skosana and colleagues, in one study, reported relatively high (90%) compliance with the South African STGs/EML, with patients in the ICU having a 97.6% compliance level [54] and 93.4% in another study [56].

The inadequacies found among the quality indicators (Table 3) suggest an urgent need to instigate ASPs in hospitals across Zambia, building on current efforts [24]. Evidence shows that ASPs, when well planned, implemented, and evaluated, lead to improvements in antibiotic use in hospital settings across Africa (Table S2). Notwithstanding the numerous structural and logistical challenges associated with implementing ASPs in LMICs, including Africa [23], there is a case to be made for well-planned, sufficiently resourced, and team-led ASPs to be implemented in Zambian hospitals building on successful approaches across Africa (Table S2) [26,29]. Moreover, an urgent need exists to enhance infection prevention and control (IPC) practices among hospitals across Zambia with concerns that excessive intubation, and the use of other invasive devices, enhance the potential for hospital-acquired infections [49,64].

We are aware of a number of limitations with our study. Data extraction may have been impacted because public hospitals in Zambia still use paper-based medical records that do not always contain complete and up-to-date information. Specific data on the antibiotic indication, disease signs and symptoms, and treatment duration are generally not clearly defined in the patient’s medical record. However, this is a drawback of all PPS studies, especially those based on paper records.

Secondly, some medical records, including prescription drug charts, are held separately from the main patient file, i.e., it was not uncommon to find drug prescription charts kept by the bedside separate from the patient file containing clinical notes, which are typically kept at the nurses’ bay. We also did not evaluate antibiotic supply data in the hospitals, which may influence their prescribing. In addition, we are aware that this study was undertaken during the recent pandemic, which may have increased the extent of antibiotic prescribing versus pre-pandemic levels [65,66,67]. Finally, the findings of this PPS were only based on 10 first- and second-level public hospitals sampled across Zambia. However, despite these limitations, we believe our findings are robust, guiding all key stakeholders going forward in Zambia and wider. This is especially the case regarding key areas of prescribing and documentation that need improving going forward.

## 4. Materials and Methods

### 4.1. Study Design and Sites

This was a cross-sectional survey adapting the WHO PPS methodology [39], which includes pre-validated data collection tools. The PPS was undertaken from 8 to 19 November 2021. Healthcare facilities of interest were the first- and second-level public hospitals among the 10 provinces in Zambia. In the Zambian health system, general hospitals are categorized as second-level referral hospitals, while the district hospitals are categorized as first-level primary healthcare hospitals, with both of these levels offering in- and out-patient healthcare services. Patients can be transferred to these facilities from local health centers or clinics. Alternatively, direct admission of mostly uncomplicated medical or surgical cases occurs. Complex cases can subsequently be referred to tertiary hospitals if needed. First and second-level hospitals also offer public health programs, including immunization programs, as well as manage patients with HIV, and offer maternal and child health services as well as screen for non-communicable diseases.

**Figure 3:**
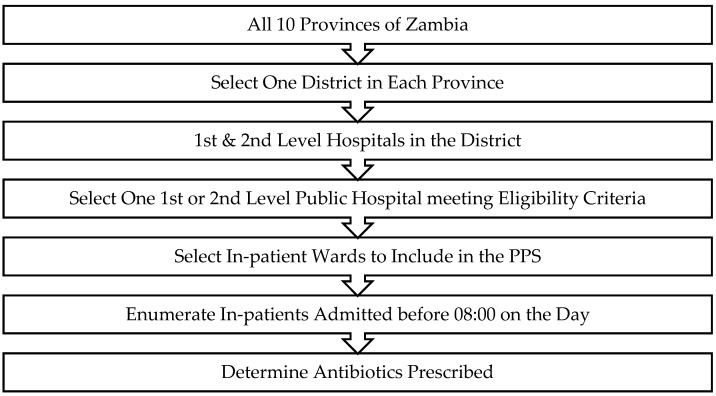
Flow plan of the PPS conducted in Zambia.

We used the WHO criteria for hospital in-patient bed capacity to select 10 first- and second-level hospitals among the 10 provinces of Zambia to participate in the survey (Supplementary Table S1). A multi-stage sampling process was followed (Figure 3) to obtain a comparable representative sample of hospitals across Zambia considering the total bed capacity and region/province using the official facility list of the Ministry of Health (https://mfl.moh.gov.zm/facility/index) (Accessed on 25 October 2021).

In Zambia, all the first- and second-level hospitals have an in-patient bed capacity below 500. Firstly, all 10 provinces in Zambia were considered. Secondly, all districts in each province were listed. Simple random sampling (raffle method) was used to select one district from each province. Thirdly, a list of all first- and second-level hospitals in each district (obtained from https://mfl.moh.gov.zm/table) (Accessed on 25 October 2021) was used to randomly (raffle method) select one hospital, i.e., either a first- or second-level hospital in that district. These 10 randomly selected public sector hospitals (Supplementary Table S1) equated to 10% of Zambia’s first- and second-level hospitals, representing the different geographical locations within the country providing similar levels of care.

All hospitals selected had the following in-patient clinical departments and wards: internal medicine, surgery (comprising general surgery), obstetrics and gynecology, pediatrics, and intensive care units (ICUs, comprising general and neonatal).

### 4.2. Patient Inclusion and Exclusion Criteria

For each selected hospital, all participants meeting the inclusion criteria were surveyed as per the WHO PPS methodology. The wards targeted for data collection among the participating hospitals were medical, surgical, obstetrics and gynecology, pediatric, and ICU wards with in-patient admissions. The medical records of all in-patients admitted to the wards of interest receiving medical treatment for at least one day, and who were admitted to the ward on or before 08:00 on the day of data collection, were included. First-level hospitals were typically surveyed in one day. Data collection typically took three days for second-level hospitals, with each ward surveyed on a single day.

Exclusion criteria included out-patients, in-patients admitted to isolation COVID-19 wards, those that did not get admitted for at least one day in the ward of interest, and in-transit discharged patients not receiving treatment. Prescriptions with topical antibiotics and anti-TB and -HIV drugs were excluded from the analysis of antibiotic prevalence patterns. However, patients with HIV, TB, or malaria prescribed antimicrobials to treat identified infections including CNS and ENT infections, as well as pneumonia or sepsis, were included in the analysis. This was in line with other PPS studies undertaken in Sub-Saharan Africa, where there can be high prevalence rates of TB, HIV, and malaria [41,49,51,54].

### 4.3. Data Collection and Analysis

Data collection for the PPS was undertaken amongst the 10 hospitals from 8 to 19 November 2021, by five teams of data collectors from the national level, with each team visiting two hospitals in different provinces. The data collectors were pharmacists who were trained intensively over a period of a week regarding the WHO-PPS methodology for data collection and data entry for analysis [39]. Teams of four data collectors per hospital extracted the required data from in-patients’ medical records with the assistance of the health staff in charge of the visited wards.

All in-patients (beds) in a single ward were completely surveyed within one day, allowing the correct calculation of the numerator (patients on antimicrobials) and the denominator (all patients in the ward). Where possible, data collection was completed for all the wards in a hospital within one day, such as among first-level hospitals with smaller bed capacity. For second-level hospitals with a relatively larger bed capacity, data collectors were allowed up to maximum three days to complete data collection from all the wards of interest at each hospital. In all cases the date of data collection was indicated on the data collection forms.

Patient data were collected from patients’ paper-based records, using pre-validated tools recommended by the WHO. None of the surveyed facilities were using electronic patient healthcare records. Three forms were used to collect specific data for the PPS in the 10 surveyed hospitals: (i) Hospital data form: general information concerning the level of the hospital, the bed capacity (divided into intensive care beds, acute beds and ordinary beds), and whether the hospital provided primary, secondary, or tertiary specialized healthcare services; (ii) Ward data form: data when the PPS was conducted and the type of ward surveyed (e.g., neonatal, female, adult or pediatric ward); and (iii) Patient data form: patients’ demographics, data on prescribed antibiotics, and whether prescribed by their generic names (INN—international non-proprietary name) or brand (originator) name, the frequency, routes of administration and whether all the prescribed doses were administered, as well as the indication and reasons of therapy. Data were collected on the site of infection and whether the infection was hospital-acquired (e.g., if a cannula or catheter was placed on the patient or whether intubation had occurred during their hospital stay) or community-acquired and whether antibiotics were given for prophylaxis or treatment. Comorbidity dates were recorded as optional information to provide an association with antibiotic use.

The prescribing and quality use indicator (QI) data collected included whether or not a microbial culture and sensitivity testing (CST) was requested, whether the indication was documented for the prescribed antibiotics, whether directed or empiric treatment, whether the start, stop or review dates of treatment were recorded, and the extent of compliance to national treatment guidelines [68]. Good compliance to agreed guidelines is increasingly used across sectors and countries as demonstrating good quality care [18,61,62]. There have been concerns with adherence rates to published guidelines among African countries [18,21,60,69,70,71,72]; however, this is not always the case [56,58,61]. In addition, the percentage of patients prescribed antibiotics by their international non-proprietary name is important to reduce costs; however, there can be concerns with their quality across countries (INN) [73,74,75,76,77].

Manually recorded data were entered on Microsoft Excel^®^ spreadsheets, collated, and cleaned. Where applicable data were coded into categories. The Anatomical Therapeutic Chemical (ATC) classification codes [78] and the WHO AWaRe antibiotic category lists were used to classify the antibiotics prescribed [34,35,36]. The final data sets were exported to Stata version 16.1 (Stata Corporation, College Station, TX, USA) for statistical analysis. Frequencies and proportions for categorical variables were calculated as a weighted percentage of antibiotic users from all the participating facilities.

### 4.4. Interpretation of Data Considering Antimicrobial Stewardship Programs

We undertook a narrative review of published papers to document a number of ASPs undertaken across Africa and their impact, including those to improve antibiotic utilization to prevent surgical site infections (Supplementary Table S2). The objective was to provide guidance to key stakeholder groups in Zambia going forward, similar to previous studies undertaken by the co-authors to stimulate activities and debates across key areas in Africa [31,79,80,81,82,83,84].

The different activities that can be undertaken when instigating ASPs are categorized according to the 4Es to enhance understanding and comparisons across situations. These include education, economics, engineering and enforcement [85]. Education incorporates activities such as developing, communicating and monitoring adherence to well-constructed guidelines [70,86,87]. Economics includes financial incentives to pharmacists, clinicians, patients or hospitals to improve the rational use of medicines, which includes incentives for clinicians when reaching agreed rational prescribing targets such as adherence to guidelines as well as fining pharmacists for dispensing an antibiotic without a prescription when this is prohibited [79,85,88]. Engineering refers to organizational or managerial interventions. This incorporates prescribing targets such as an agreed percentage of antibiotics being prescribed according to current guidelines or according to the WHO AWaRe list and the percentage of patients prescribed short courses of antibiotics to prevent SSIs [31,34,41,89]. Enforcement entails enforcing regulations by law, including prohibiting the dispensing of antibiotics within pharmacies or elsewhere without a prescription [90,91].

### 4.5. Ethical Considerations

This PPS study was part of an ongoing national program by the Zambian Ministry of Health to improve the rational use of medicines within public hospitals across the country [41,46,54,56,92,93,94]. Consent and official permission for the survey were granted by the Ministry of Health headquarters through the Department of Clinical Care and Diagnostic Services. The survey did not involve interventions or interactions with patients, with all information collected retrospectively from patient’s medical records and notes. Site permissions from each surveyed hospital were obtained beforehand to access and collect data from the medical records of pertinent patients. The information was de-identified with no personal details of the patients involved in the survey subsequently documented. Each medical record was assigned a reference number for checking purposes, with all data collected kept confidentially.

## 5. Conclusions

The combined prevalence of antibiotic use among the 10 surveyed public first- and second-level hospitals across Zambia was higher than the threshold recommended by WHO; however, some utilization rates among the surveyed hospitals were lower than this, mirroring a number of hospitals across Africa. Antibiotics were mostly prescribed empirically, with ceftriaxone—a ‘Watch’ antibiotic—the most prescribed. This needs to be urgently addressed where pertinent in order to reduce the resistance potential given rising rates of AMR within Zambia.

There were also concerns with the low use of CST to guide antibiotic choices, poor recording of the rationale for the chosen antibiotic in patients’ notes, and limited documentation of a stop date. These are also key areas to address to improve future prescribing of antibiotics within hospitals in Zambia, and we will now be looking to instigate educational and other programs among hospitals in Zambia. Alongside this, current low compliance rates to national STGs among the participating hospitals also need to be urgently addressed as this is a key quality target. The instigation of appropriate ASPs will be key to optimizing future antibiotic use in Zambia and reducing current AMR rates, and we will be following up on this in future projects.

## Figures and Tables

**Table 1:** Demographics of patients prescribed antibiotics across all surveyed hospitals (*n* = 307).

Variable	Level	Patients, *n* (%)
Hospital facility	Hospital 1 Hospital 2 Hospital 3 Hospital 4 Hospital 5 Hospital 6 Hospital 7 Hospital 8 Hospital 9 Hospital 10	31 (10.1) 27 (8.8) 16 (5.2) 60 (19.5) 65 (21.2) 36 (11.7) 6 (2.0) 7 (2.3) 7 (2.3) 52 (17.0)
Ward specialty	Adult Medical Ward Adult Surgical Ward Mixed Adult Ward Mixed Pediatric Ward Neonatal Intensive Care Unit Pediatrics Medical Ward Not recorded	81 (27.9) 21 (7.2) 87 (30.0) 27 (9.3) 20 (6.9) 54 (18.6) 17 (5.6)
Gender	Female Male Not recorded	163 (53.1) 139 (45.3) 5 (1.6)
Age (years)	<5 ≥5 Not recorded	76 (24.8) 220 (71.7) 11 (3.5)
Central vascular catheter	Yes No Not recorded	1 (0.3) 275 (89.6) 31 (10.1)
Urinary catheter	Yes No Not recorded	59 (19.2) 210 (68.4) 38 (12.4)
Peripheral vascular catheter	Yes No Not recorded	247 (80.5) 45 (14.7) 15 (4.9)
Intubation	Yes No Not recorded	6 (2.0) 238 (77.5) 63 (20.5)
Malaria status	Yes No Unknown/not recorded	28 (9.1) 112 (36.5) 167 (54.4)
HIV status	Yes No Unknown/not recorded	36 (11.7) 75 (24.4) 196 (63.9)
HIV on Antiretroviral Therapy	Yes No Unknown/not recorded	24 (7.8) 73 (23.8) 210 (68.4)
Tuberculosis status	Yes No Unknown/not recorded	9 (2.9) 99 (32.2) 199 (64.8)

**Table 2:** Antibiotics prescribed, their mechanism of action, and common infections treated in the surveyed hospitals.

Variable and *n*	Infection/Antibiotics Prescribed	Frequency, *n* (%)
Diagnosis, *n* = 307	CNS	9 (2.9)
	CVS	6 (2.0)
	ENT	17 (5.5)
	GI	23 (7.5)
	OBGY	38 (12.4)
	Pneu	36 (11.7)
	Sepsis	13 (4.2)
	Other	165 (53.7)
Antibiotic (ATC code/and AWaRe classification) and Mode of action, *n* = 534	**Access**	
	J01CA04 (Amoxicillin)—Bactericidal; inhibits bacterial cell wall biosynthesis	27 (5.1)
	J01CE01(Benzyl Penicillin)—Bactericidal; inhibits bacterial cell wall biosynthesis	43 (8.1)
	J01CF02 (Cloxacillin)—Bactericidal; inhibits bacterial cell wall biosynthesis	29 (5.4)
	J01GB03 (Gentamicin)—Bactericidal; interferes with bacterial protein synthesis	25 (4.7)
	P01AB01 (Metronidazole)—Bactericidal; disrupts bacterial DNA synthesis	58 (10.9)
	**Watch**	
	J01DD01 (Cefotaxime)—Bactericidal; inhibits bacterial cell wall biosynthesis	70 (13.1)
	J01DD04 (Ceftriaxone)—Bactericidal; inhibits bacterial cell wall biosynthesis	193 (36.1)
	JO1MA02 (Ciprofloxacin)—Bactericidal; inhibits bacterial DNA gyrase enzyme	30 (5.6)
	Others *	59 (11.1)
Parenteral type, *n* = 307	IM	4 (1.3)
	IV—Bolus	205 (66.8)
	IV—Continuous (via a catheter)	29 (9.4)
	Other	69 (22.5)

Key: CNS: central nervous system; CVS: cardiovascular system; ENT: ear nose throat; GI: gastrointestinal, OBGY: obstetrics and gynecology; Pneu: pneumonia. * Other antibiotics prescribed included ampicillin (bactericidal; inhibits bacterial cell wall biosynthesis), azithromycin (bactericidal; interferes with bacterial protein synthesis), co-trimoxazole (bactericidal; interferes with bacterial protein synthesis), erythromycin (bactericidal; interferes with bacterial protein synthesis), meropenem (bactericidal; inhibits bacterial cell wall biosynthesis), nitrofurantoin (bactericidal; disrupts bacterial DNA, RNA, and protein synthesis), and penicillin-V (bactericidal; inhibits bacterial cell wall biosynthesis). IM: intramuscular; IV: intravenous.

**Table 3:** Quality indicator measures for antibiotics prescribed (*n* = 534).

Variable	% Undertaken/Adherence
Rationale for prescription documented in the patient’s notes/indication documented in patient’s notes	15.7%
CST requested	3%
% Empiric use *	97%
Stop/review data stated in the patient’s notes	32%
Compliance with Zambian STGs	27%
INN prescribing	83%

NB: CST: culture and sensitivity testing; * absence of CST; INN: International Non-Proprietary Name.

## Data Availability

Data are available from the corresponding authors on reasonable request.

## Supplementary Materials

The following supporting information can be downloaded at: https://www.mdpi.com/article/10.3390/antibiotics11111626/s1, Table S1: Hospital Facilities Selected by Province. Table S2—Examples of ASPs successively introduced across Africa to improve antimicrobial prescribing. This includes [95,96,97,98].
