# Supplementary material for: Antibiotic Use and Stewardship Indicators in the First- and Second-Level Hospitals in Zambia: Findings and Implications for the Future
Source: Antibiotics (Basel). 2022 Nov 15;11(11):1626. doi: 10.3390/antibiotics11111626 (PMC9687079; doi:10.3390/antibiotics11111626)
Supplement: Supplementary file 1 [file antibiotics-11-01626-s001.zip › antibiotics-1967764-supplementary.pdf]

Supplementary Tables

Table S1: Hospital Facilities Selected by Province

Province	District	Hospital Facility Name
Lusaka Province	Chongwe	Chongwe District Hospital
Southern Province	Monze	Monze Mission Hospital
Western Province	Senanga	Senanga District Hospital
North-western Province	Solwezi	Solwezi General Hospital
Copperbelt Province	Luanshya	Roan General Hospital
Central Province	Serenje	Serenje District Hospital
Muchinga Province	Chinsali	Chinsali General Hospital
Northern Province	Kasama	Kasama General Hospital
Eastern Province	Petauke	Kalindawalo General Hospital
Luapula Province	Samfya	Samfya District Hospital

Table S2 – Examples of ASPs successively introduced across Africa to improve antimicrobial prescribing

Author, country and year	Brief details of the intervention	Impact of the Intervention
Aitken et al, Kenya, 2013 [95]	Education and Engineering to develop, implement and monitor a policy within the hospital to reduce excessive post-operative prescribing of antibiotics to reduce SSIs	• Appreciable improvement in reducing post-operative prescribing of antibiotics to 40% of operations within the first week and just 10% by week 6 following policy implementation (p< 0.0001)<br>• Overall, net reduction in the costs for IV antibiotics and associated consumables used to prevent SSIs at approximately US$2.50/operation
Ntumba et al, Kenya, 2015 [71]	Education and Engineering to reduce excessive prescribing of antibiotics post-operatively to prevent SSIs. Documented activities included:<br>• Local adaptation of published guidelines<br>• Creation and tools for advocacy, training, and leadership around appropriate antibiotic use to prevent SSIs	• Patients receiving antibiotics post-operatively decreased from 50% to 26%<br>• Alongside this, crude SSI rates significantly decreased from 9.3% to 5% of patients
Allegranzi et al, Kenya, Uganda, Zambia, and Zimbabwe, 2018 [33]	Education and Engineering to reduce inappropriate (post-operative) prescribing of antibiotics to reduce SSIs. Documented activities included:<br>• Five planned visits to each participating hospital among four African countries during the study period - supported by a range of educational tools<br>• Local teams identified key areas of concern with preventing SSIs; subsequently monitoring an agreed range of indicators (six pre-identified ones including skin preparation and optimal timing of prophylaxis)<br>• Subsequent launch of pertinent tools and agreed indicators alongside monitoring/ feedback to improve future prescribing	• Appropriate use of antibiotics to prevent SSIs improved from 12.8% (baseline) to 39.1% of patients (p <0·0001) among the studied hospitals<br>• Concurrently, the cumulative incidence of SSIs decreased from a baseline of 8.0% to 3.8% post intervention (p <0·0001)
Alabi et al, Liberia, 2022 [96]	• Education and Engineering combining three key activities - the production and dissemination of local treatment guidelines, training and regular AMS ward rounds as well as monitoring adherence to agreed quality indicators (QIs)<br>• QIs included prescribing of correct antibiotics (incorporating completeness of	Improvements were seen in all QIs:<br>• Adherence to local guidelines improved from 34.5% to 61.0% (P<0.0005)<br>• Correct dosing improved from 15.2% to 36.5% of antibiotics prescribed (P<0.0005)<br>• Optimal duration of antibiotic use improved from 13.2% to 31.0% (P<0.0005)

	microbiological diagnostics) as well as their dosages and duration<br>• QIs were assessed in a case series after AMS ward rounds and fed back to key personnel to further improve their antibiotic prescribing	• Proportion of patients receiving ceftriaxone reduced from 51.3% to 14.2% (P<0.0005).<br>• Following the ASP, 79.7% of patients now have samples sent for microbiological analysis
Lester et al, Malawi, 2020 [30]	• Education and Engineering involving guidelines, posters and the application of smartphones to help with clinical decision making as well as regular PPS studies combined with prescriber feedback<br>• The objective was to reduce extensive prescribing of third-generation cephalosporins within the hospital and associated costs alongside no adverse impact on mortality – especially with high rates of HIV among in-patients in the hospital (approximately 61% across the surveys)	• The proportion of prescriptions for an IV 3rd generation cephalosporin fell from 80.1% of all prescriptions at the first survey to 53.6% by the last survey<br>• The median length of a ceftriaxone course of treatment was reduced from 5 to 4 days - aided by an increase in the number of clinician reviews of prescriptions at 48-hours which increased from 22.4% at the start to 73.3% by the final antibiotic survey<br>• Annual savings were estimated at US$15,000 with no change in mortality or median length of hospital stay
Brink et al., South Africa, 2016 [28]	Principally Education. Documented activities involved:<br>• Initial training sessions with key stakeholders in each hospital discussing the five process measures that would subsequently be audited by pharmacists in each hospital were – this was provided through face-to-face regional learning sessions<br>• Subsequently each pharmacist was required to undertake audits of the five measures in their respective hospitals<br>• The five measures included: Cultures not performed before starting empiric treatment; prolonged treatment (7 and 14 days); more than 4 antibiotics concurrently; concurrent double or redundant antibiotic coverage	• Reduction in mean antibiotic defined daily doses/ 100 patient days from 101·38 to 83·04 (p<0·0001)<br>• Reductions also in the number of cultures not performed before starting empiric treatment, prolonged antibiotic treatment (7 and 14 days), more than 4 antibiotics prescribed concurrently, and the prescribing of concurrent double or redundant antibiotic coverage among the participating hospitals
Boyles et al., South Africa, 2017 [32]	Education and Engineering to improve future antibiotic use in the hospital. Key activities included:<br>• A comprehensive ASP programme comprising online education, a dedicated antibiotic prescription chart and weekly dedicated ward rounds to discuss current prescribing – continued over 4 years<br>• Pre- and post-intervention data compared	• Total antibiotic consumption fell from 1,046 defined daily doses/1 000 patient days (pre-intervention) to 868 (first 2 years of the intervention - remaining at similar levels for the next 2 years)<br>• Improvements driven by reductions in IV antibiotic use, particularly ceftriaxone<br>• Laboratory testing increased over the same period<br>• Cost savings on antibiotics (inflation adjusted) were ZAR3.2 million over 4 years

		• No significant change in mortality or 30-day readmission rates over the 4 years
van den Bergh et al, South Africa, 2020 [27]	Principally education to improve compliance to agreed guidelines for Community Acquired Pneumonia (CAP):<br>• A CAP bundle was developed which incorporated seven process measures, which included admission criteria, antibiotic choices, dose and length, as well as three outcome measures including length of hospital stay and mortality, which pharmacists subsequently used to audit compliance to the bundle and provide feedback<br>• Training sessions were conducted on the CAP guidelines and implementing ASPs within hospitals across South Africa. Following each learning session, a checklist of essential activities and deadlines was provided to each attending pharmacist<br>• Baseline data was collected to identify areas for improvement<br>• In a four-week period following the learning sessions, pharmacists subsequently applied the learnt ideas to improve compliance to the CAP guidelines and ways to give feedback to address identified gaps to further improve future compliance	2464 patients from 39 hospitals were included with the ASP showing positive results:<br>• CAP bundle compliance improved from 47.8% to 53.6% ($p < 0 \cdot 0\ 0\ 01$)<br>• Diagnostic stewardship compliance improved from 49.1% to 54.6% ($p < 0 \cdot 0\ 0\ 01$)<br>• Improved compliance with process measures was significant for 5 of the 7 components, which included choice and dose of antibiotics prescribed as well as IV to oral switching<br>• However, there was no significant difference in mortality or median length of stay pre- and post-intervention<br>•
Bashar et al, South Africa, 2021 [97]	• Education and Engineering involving regular ASP ward rounds on two surgical wards<br>• During the ward rounds - each condition was discussed especially concerning antibiotic selection and laboratory investigations<br>• In addition, potential switching from intravenous to oral agents, dose optimisation and any dose adjustments in patients with renal and hepatic impairment	• Reduction in the volume of antibiotic consumption from 739.30 DDDs/1000 to 564.93 DDDs/1000 patient days following the ASP<br>• Reduction in inappropriate antibiotic use from 35% to 26%<br>• An overall increase in culture targeted therapy<br>• Reduction in antibiotic administration for more than one day post operatively to prevent SSIs (from 7.3% to 6.6%)<br>• Small (non-significant reduction) in total antibiotics administered IV (from 89.4% to 84.2%) alongside an increase in appropriate IV administration from 56.9% to 60.8%
Ngonzi et al, Uganda, 2021 [98]	• Principally Education and Engineering regarding World Health Organization's checklist of activities to reduce SSIs (WHO	• The use of the WHO's SSC increased from 7% pre-intervention to 92% in the

	SSC) in patients undergoing caesarean sections<br>• This involved educational interventions combined with daily audits and feedback to improve antibiotic prescribing	intervention phase (P < 0.001) – then fell to 77% post-intervention (P < 0.001)<br>• Prescribing of antibiotics rose from 18% of patients pre-intervention to 90% in the intervention phase (P < 0.001); subsequently 84% post-intervention phase (P < 0.001)<br>• Documented SSI rate fell from 15% pre-intervention phase to 7% in the intervention phase (P = 0.02)
